# Hepatic Myofibroblasts: A Heterogeneous and Redox-Modulated Cell Population in Liver Fibrogenesis

**DOI:** 10.3390/antiox11071278

**Published:** 2022-06-28

**Authors:** Claudia Bocca, Francesca Protopapa, Beatrice Foglia, Marina Maggiora, Stefania Cannito, Maurizio Parola, Erica Novo

**Affiliations:** Unit of Experimental Medicine and Clinical Pathology, Department of Clinical & Biological Sciences, University of Torino, 10125 Torino, Italy; claudia.bocca@unito.it (C.B.); francesca.protopapa@unito.it (F.P.); beatrice.foglia@unito.it (B.F.); marina.maggiora@unito.it (M.M.); stefania.cannito@unito.it (S.C.); maurizio.parola@unito.it (M.P.)

**Keywords:** hepatic myofibroblasts, liver fibrogenesis, reactive oxygen species, oxidative stress, chronic liver diseases

## Abstract

During chronic liver disease (CLD) progression, hepatic myofibroblasts (MFs) represent a unique cellular phenotype that plays a critical role in driving liver fibrogenesis and then fibrosis. Although they could originate from different cell types, MFs exhibit a rather common pattern of pro-fibrogenic phenotypic responses, which are mostly elicited or sustained both by oxidative stress and reactive oxygen species (ROS) and several mediators (including growth factors, cytokines, chemokines, and others) that often operate through the up-regulation of the intracellular generation of ROS. In the present review, we will offer an overview of the role of MFs in the fibrogenic progression of CLD from different etiologies by focusing our attention on the direct or indirect role of ROS and, more generally, oxidative stress in regulating MF-related phenotypic responses. Moreover, this review has the purpose of illustrating the real complexity of the ROS modulation during CLD progression. The reader will have to keep in mind that a number of issues are able to affect the behavior of the cells involved: a) the different concentrations of reactive species, b) the intrinsic state of the target cells, as well as c) the presence of different growth factors, cytokines, and other mediators in the extracellular microenvironment or of other cellular sources of ROS.

## 1. Introduction: Role of Hepatic Myofibroblasts in the Scenario of Liver Fibrogenesis

Hepatic myofibroblasts (hMFs) represent a heterogeneous population of α-smooth muscle actin (α-SMA)—positive liver cells playing a critical pro-fibrogenic role in the progression of chronic liver disease (CLD). CLDs are characterized by a long-standing history of parenchymal injury (on average 15–20 years) resulting in the persistent activation of inflammatory and fibrogenic or wound healing responses. In this review, the definition of liver fibrogenesis will be used to indicate a highly dynamic and integrated molecular, cellular, and tissue process that, with time, can lead to liver fibrosis, intended as an excessive accumulation of extracellular matrix (ECM) components in liver parenchyma [1,2,3,4,5,6,7,8,9]. As it is well known, fibrogenesis and fibrosis are critical features of CLD progression to more advanced stages of the disease leading eventually to liver cirrhosis, a stage characterized by the derangement of liver architecture typically involving the formation of regenerative nodules of parenchyma surrounded by fibrotic septa. Moreover, significant vascular changes are typical of cirrhosis, being responsible for portal hypertension and related clinical complications (variceal bleeding, hepatic encephalopathy, ascites, hepatorenal syndrome, etc.) [10]. Changes in vascular architecture found in chronically injured livers are intimately linked to the parallel development of fibrogenesis and pathological angiogenesis, two processes that are believed to strongly affect each other, in which MFs play a critical role [11,12,13]. Finally, patients experiencing CLD progression have a significantly increased risk of developing hepatocellular carcinoma (HCC), the most frequent primary liver cancer (70–90%) that now represents the fourth leading cause of cancer mortality worldwide [14,15,16].

In the scenario of CLDs, MFs can originate from different cellular sources following activation by a number of mediators and conditions, including reactive oxygen species (ROS) and other oxidative stress-related mediators [17,18,19,20,21].

Interestingly, regardless of the cellular origin, MFs exhibit rather common phenotypic responses including not only the ability to produce and release an excess of ECM components (i.e., leading with the time to liver fibrosis) but also a high proliferative attitude and the ability to respond to—as well as to release—several mediators in a scenario dominated by chronic liver injury [17,18,19,20,21]. This peculiar MF phenotype has been detected in all the major forms of progressive CLD, including conditions related to chronic infection by hepatotropic viruses such as HBV and HCV, excessive consumption of alcohol (i.e., leading to alcoholic liver disease or ALD), metabolic derangement such as in the case of non-alcoholic fatty liver disease (NAFLD, often detected in obese and/or diabetes type 2 patients), autoimmune hepatitis type I and II, and in hereditary diseases involving the liver such as hereditary hemochromatosis, α1-antitrypsin disease, and Wilson’s disease [1,2,3,4,5,6,7,8,9,17,18,19,20,21]. In these conditions, inflammation and several mediators released from injured hepatocytes, including ROS, lead to MFs activation. This event is also associated with a decrease in antioxidant defenses particularly evident in HCV and HBV infections in which the accumulation of ROS sustains cellular and tissue damage.

In particular, the prevalent profibrogenic mechanism in HBV- or HCV-related CLD progression is represented by chronic activation of wound healing, with ROS and oxidative stress also offering a relevant contribution [8].

However, liver MFs also play a critical role in cholangiopathies, either autoimmune-mediated such as primary biliary cholangitis and primary sclerosing cholangitis, as well as other rare genetically related conditions (Alagille syndrome, Caroli syndrome, ABCB4 deficiency, cystic fibrosis, polycystic disease), or the so-defined idiopathic cholangiopathies (biliary atresia, sarcoidosis) [22,23,24].

Since it is well known that oxidative stress plays a key role in driving liver damage and the initiation/progression of liver fibrosis, in the present review, we will focus our attention on the behavior of liver MFs in response to ROS and, more generally, on the ability of oxidative stress in modulating MF-related phenotypic responses.

## 2. MFs Involvement in the Scenario of Liver Fibrogenesis

### 2.1. Pro-Fibrogenic Cells and Mediators

In the presence of an etiological agent or condition eliciting persistent hepatocellular/parenchymal injury, fibrogenesis is unequivocally fueled by the chronic inflammatory response and is related to intense cross-talk between different cell populations either resident in the liver or extrahepatic [1,2,3,4,5,6,7,8,25,26,27]. The hepatic cells involved are injured hepatocytes, defenestrated sinusoidal endothelial cells (SECs), activated hepatic stellate cells (HSCs), and other cellular sources of liver MFs, Kupffer cells (KCs), hepatic progenitor cells, and cholangiocytes. Other cells that significantly contribute to the fibrogenic scenario are innate immunity mononuclear cells recruited from peripheral blood and activated to multiple macrophage phenotypes in the injured liver [25,26,27]. An additional role is also played by other immune cells such as T and B lymphocytes, natural killer (NK) cells, as well as natural killer T (NKT) cells [1,2,3,4,5,6,7,8].

**Figure 1:**
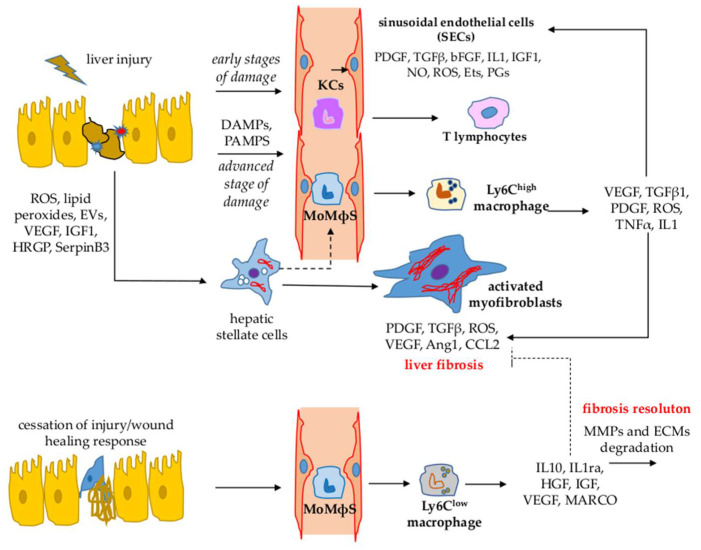
Most relevant interactions between the major cell populations involved in CLD. MoMΦs: monocyte-derived macrophages.

All these cell types communicate with each other by either releasing, in a paracrine and/or autocrine way, or responding to a plethora of mediators including growth factors, inflammatory cytokines, chemokines, adipokines, endothelins, components of the renin/angiotensin systems and other plasma proteins, ROS and other oxidative stress–related reactive molecules, pathogen-associated or damage-associated molecular patterns (PAMPs and DAMPs, respectively), as well as agents/compounds acting as ligands for the pattern-recognition receptor (PRR) [3,4,7,8,21]. Figure 1 represents a summary of the most relevant interactions between the main cellular populations involved in CLD progression.

### 2.2. Hepatic MFs: A Heterogeneous Population of Pro-Fibrogenic Cells in CLD Progression

Liver MFs can originate from the activation of different cellular precursors of mesenchymal origin [1,2,3,4,5,6,7,8,9,10,11,12,13,17,18,19,21] (Table 1) represented by:

(a) HSCs, the major source of hepatic MFs [1,2,3,4,5,6,7,8,9,17,18,19,21,28,29,30].

Physiologically, HSCs reside in the space of Disse where they make direct contact with hepatocytes, SECs, and other HSCs; these cells are considered responsible for the deposition of ECM and also play a role as liver pericytes and in vitamin A and retinoids storage/metabolism [17]. When HSCs become activated to MF-like cells (sometimes indicated with the acronym HSC/MFs, to make clear that they are MFs derived from HSC activation), these essential functions are lost or deeply modified. HSC/MFs are extremely well characterized in terms of (i) markers that can be used to identify them in liver specimens, including glial fibrillary acidic protein or GFAP, platelet-derived growth factor (PDGF) receptor β or PDGFRβ, nerve growth factor receptor p75 subunit, lecithin-retinol acyltransferase or LRAT, integrin ανβ3, vimentin, desmin, mannose 6-phosphate/insulin-like growth factor II receptor, and cytoglobin [17,21,31]; and (ii) genome-wide transcriptome profiling, which has revealed an impressive number of HSC-specific genes and HSC/MFs-specific gene signatures, with the latter proposed to be associated with poor patient prognosis [32];

(b) Portal fibroblasts, the second major cellular source of hepatic MFs.

These cells consist of a population of mesenchymal cells residing in the connective tissue of portal areas, positive for α-SMA, as well as for a specific marker such as ecto-ATPase nucleoside triphosphate diphosphohydrolase-2 (NTPD2) and others less specific such as fibulin 2, elastin, IL-6m and cofilin 1 [30,33]. The real numerical contribution of MFs derived from portal fibroblasts to CLD progression is still debated [28,29] but there is no doubt that they are present in almost all CLDs. There is a consensus on the fact that portal MFs may predominate in conditions of biliary fibrosis and, accordingly, portal fibroblasts may be the earliest cell population of mesenchymal origin activated following specific injury to biliary epithelial cells or cholangiocytes [34,35];

(c) Cells originating in the bone marrow and recruited into chronically injured liver.

Initially, this was reported for a selected cohort of female patients developing HCV-related CLD after receiving a bone marrow transplant from male donors in which a significant percentage of MFs was positive for the Y chromosome [36]. Then the origin of bone-marrow-derived cells was confirmed by experimental studies suggesting mesenchymal stem cells [37,38] or α-SMA negative precursor cells [39] as cell precursors. However, at present, the overall consensus is that MFs from bone-marrow-derived cells represent just a minority of hepatic MFs detected in CLDs.

In the past, epithelial cells, hepatocytes, and cholangiocytes were also considered a potential source of liver MFs through a process of the epithelial-to-mesenchymal transition (EMT), mainly supported by studies in lung and kidney fibrosis [7], but at the moment, the involvement of EMT in CLDs as a source of MFs is controversial and, in any case, considered to be of minor relevance [19,20,28,30,40,41].

### 2.3. Activation and Major Phenotypic Responses of Liver MFs

**Figure 2:**
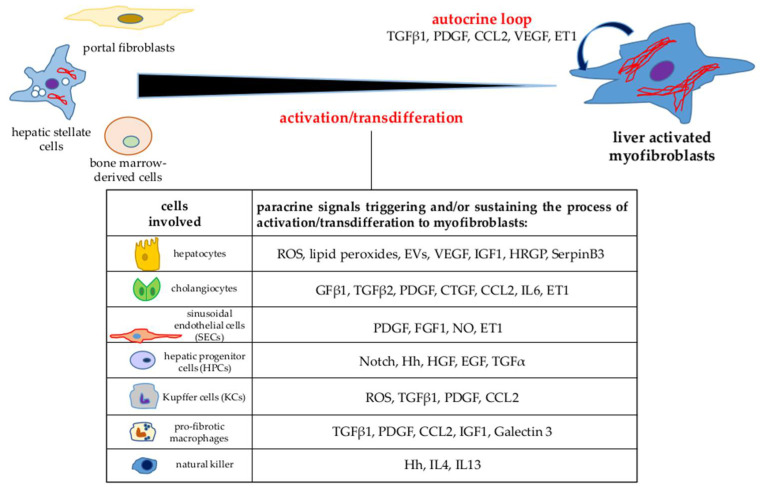
Paracrine and autocrine signals released by hepatic cell populations able to elicit and/or sustain the activation/transdifferentiation process of liver myofibroblasts.

During CLD, MFs undergo a process of activation/transdifferentiation, which is elicited and/or sustained by a long list of paracrine and/or autocrine signals [3,8,17,18,19,21], including cytokines, chemokines, growth factors, ROS, adipokines, and several other mediators released by either hepatic or extrahepatic cells infiltrating the injured parenchyma (innate and adaptive immune cells, bone marrow-derived cells) (see Figure 2). In particular, HSCs activation into MFs can be promoted by extracellular events, including persistent epithelial cell injury (whatever the cause/etiology), altered ECM, immune regulation, metabolic dysregulation, and enteric dysbiosis. Moreover, the process of HSC activation has been related to molecular dysregulation, involving membrane receptor and nuclear receptor signaling pathways, transcription factors, epigenetic transcriptional deregulation, and dysregulation of cellular homeostasis. Then, when activated, MFs can contribute to CLD progression by releasing additional mediators and signals. However, liver MFs can be easily identified in liver biopsies or specimens for their immune positivity to α-SMA and essentially share common characteristics and phenotypic responses [1,2,3,4,5,6,7,8,9,17,18,19,21] that all concur to fibrogenic disease progression and that are briefly described below [1,2,3,6,7,8,9,17,18,21,42]:

- Synthesis of ECM components. In progressive CLD, liver MFs become able to increase the synthesis of ECM components; in particular, these cells up-regulate the transcription and deposition of fibrillar collagen, mainly collagen type I and III, as well as laminin, fibronectin, and α-SMA. The synthesis of these ECM components is stimulated by several pro-fibrogenic growth factors and mediators, in particular TGFβ1 (mainly released by activated macrophages and MFs), ROS, and other oxidative stress-related mediators. Moreover, liver MFs are also characterized by a dysregulation of the genes coding for enzymes involved in ECM remodeling that leads to up-regulation of the expression of tissue inhibitors of metalloproteases (TIMPs, particularly TIMP1 and TIMP2) and down-regulation of metalloproteases with consequent insufficient removal of fibrillar collagen.
- Proliferation and survival of MFs. Liver MFs are highly proliferative cells in response to mitogenic signals, released in the pro-fibrogenic scenario by almost all cell types involved. The most potent mitogen for activated HSCs and liver MFs is PDGF released by macrophages, MFs, and SECs. PDGF exerts its action since MFs overexpress the α- and β-receptor subunit (i.e., PDGF-Rα and PDGF-Rβ). Many other stimuli and mediators are able to stimulate MFs proliferation and survival such as TGFα, epidermal growth factor (EGF), connective tissue growth factor (CTGF), thrombin, basic fibroblast growth factor (bFGF), and leptin. Moreover, persistently activated HSC/MFs have been reported to survive the induction of apoptosis in response to different agents or conditions, including high levels of ROS, due to increased expression of Bcl-2 and up-regulation of PI3K/c-Akt signaling [43,44].
- MFs migration. In progressive CLD, the ability of MFs to migrate and align along the nascent fibrotic septa in response to different chemoattractants (including at least PDGF, CCL2, VEGF-A, and Oncostatin M) and in a redox-dependent manner plays a key role.
- MFs as pro-inflammatory cells. By releasing cytokines, interleukins, and chemokines, activated hepatic MFs exert a significant pro-inflammatory role. In particular, they release the chemokines CCL2 and CCL21 able to recruit monocytes from peripheral blood or act on either T lymphocytes or activated T lymphocytes. Moreover, literature data reported the activation of the NLRP3 inflammasome not only in macrophages but also in liver MFs, which then may actively contribute to fibrogenic progression by also up-regulating IL-1β release.
- MFs as pro-angiogenic cells. Liver MFs have an active role in pathological angiogenesis detected in CLD progression. In particular, HSC/MFs are able to respond to hypoxic conditions, which develop progressively in a chronically injured liver, by up-regulating the expression and the release of key pro-angiogenic mediators, including VEGF-A, Angiopoietin-1, hedgehog ligands, and PDGF-BB, as well as up-regulating the synthesis of cognate receptors for these pro-angiogenic factors. Since angiogenesis usually precedes or accompanies fibrogenesis, it has been proposed that hypoxia may also serve to drive both processes, with HSC/MFs then representing a critical cellular crossroad by their ability to contribute to both ECM deposition and angiogenesis [8,9,13,45,46].
- MFs and CLD progression. Liver MFs can critically contribute to the perpetuation of liver fibrogenesis through their ability to establish autocrine/paracrine loops: Mediator-stimulated MFs up-regulate the expression of critical growth factors, cytokines, chemokines, and other mediators (such as TGFβ1, PDGF, CCL2, VEGF, endothelin-1, or ET-1) that, in turn, when released in the extracellular environment, can act on surrounding cells, including MFs themselves [1,2,3,4,13,16,17,18,21].

### 2.4. Pro-Fibrogenic Mechanisms and Related Issues

**Figure 3:**
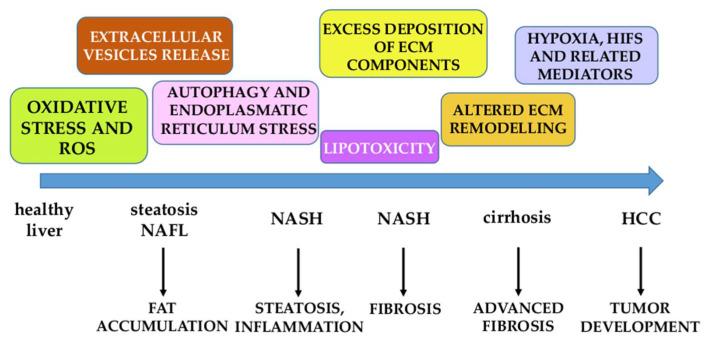
Major mechanisms involved in NAFLD progression. NAFL: non-alcoholic fatty liver; NASH: non-alcoholic steatohepatitis; HCC: hepatocarcinoma.

Chronic parenchymal injury, persisting activation of the inflammatory response, and sustained activation of wound healing and repair responses represent the main issues driving liver fibrogenesis. CLD fibrogenic progression is the result of several pro-fibrogenic mechanisms that should be classified into two categories: (i) Mechanisms that can be referred to as common and etiology independent, being detected in all forms of CLD; (ii) and etiology-related mechanisms and issues more properly and intimately related to the specific etiology of the CLD considered [8,9]. Below, the reader will find a short list of major mechanisms considered relevant for CLD progression (Figure 3):

- Oxidative stress and ROS are so relevant that we will dedicate most of the remaining sections in this review to analyzing and discussing the most critical related issues.
- Excess deposition of ECM components, mainly fibrillar collagen type I and III, is associated with qualitative changes in their topographical distribution. The altered ECM remodeling observed is due to up-regulation of the expression of TIMPs and MMPs accompanied by the non-efficient removal of fibrillar collagen [1,5,6,7,8,9].
- Hypoxia, HIFs, and related mediators are considered major determinants for fibrogenic progression and likely also for the development of hepatocellular carcinoma [47].
- Autophagy and endoplasmic reticulum stress are mechanisms involved in the activation of HSCs into HSC/MFs, with the inositol-requiring enzyme 1α (IRE1α) and PKR-like endoplasmic reticulum kinase (PERK) pathways playing a pro-fibrogenic role [48,49].
- Extracellular vesicles (EVs) are particles of different sizes released by injured or apoptotic hepatocytes in different conditions of CLDs. EVs can mediate pro-inflammatory, pro-angiogenic, and pro-fibrogenic signals since they contain miRNAs, mRNAs, signaling proteins, and lipids, potentially able to affect all surrounding cells [50,51,52,53].
- During NAFLD progression, in ALD and likely in one-third of all HCV patients that develop steatosis and steatohepatitis, lipotoxicity is believed to be responsible for hepatocyte injury and associated with nutrient/caloric overload, as well as dysfunctional adipose tissue and gut–liver axis dysbiosis [54,55,56,57].
- In the last decade, a number of genetic variants were identified as relevant risk factors for NAFLD and ALD progression, some of them even for HCC development. The most relevant genetic variants are represented by (i) patatin-like phospholipase domain containing-3 (PNPLA3) gene; (ii) transmembrane 6 superfamily member 2 (TM6SF2) gene; (iii) membrane-bound O-acyltransferase domain-containing 7 (MBOAT7), and transmembrane channel-like 4 (TMC4) genes [58,59,60].

All mechanisms described represent the basic knowledge for developing novel strategies and approaches to targeted therapies designed to affect CLD progression and have actually been tested in clinical trials.

## 3. Liver Fibrosis as a Potentially Reversible Event

Literature data published in the last two decades from pre-clinical and clinical studies have now established that fibrosis is potentially a reversible event [61]. More precisely, one should consider that fibrosis and sometimes even cirrhosis can undergo either resolution or at least regression. These two terms, although both positive for a CLD patient, should be distinguished: (i) The term resolution refers to an ideal condition, still at an early stage of the CLD and then of early fibrosis, in which a progressive removal of the excess deposition of ECM and the functional recovery of the organ can be accomplished; (ii) the term regression, which can be more properly applied to conditions of advanced fibrosis or cirrhosis, indicates, in turn, a reduction of fibrosis and only a partial functional recovery. Both events, resolution or regression, can occur only when the etiological agent or condition causing chronic liver injury is either eliminated or significantly limited [61].

In this contest, monocyte-derived macrophages (MoMΦs) recruited from peripheral blood play a key role [25,26,27] as they can differentiate into different phenotypes, depending on the mediators present in the microenvironment:

(i). The Ly6C^high^ phenotype, mostly dependent on chemoattractants (CCL2, CCL1, and CCL25) released by activated KCs, and activated HSCs and SECs [61,62,63,64]. Ly6C^high^ macrophages exert a pro-angiogenic, pro-inflammatory, and pro-fibrogenic role by releasing mediators, including TGFβ1, PDGF, and VEGF-A, able to contribute to hepatic MFs activation [1,2,3,8,9] as well as to enhance their survival in an NF-kB-dependent way [1,25,26,27].
(ii). The Ly6C^low^ phenotype (positive for markers such as Arginase-1, Arginase-2, CD206, and CX3CR1) is characterized by the increased expression and release of IL-10 and the IL-1 receptor antagonist (IL-1ra), as well as the hepatocyte growth factor (HGF), insulin-like growth factor (IGF) and VEGF-A and phagocytosis-related genes such as the alveolar macrophage marker gene (MARCO) [25,26,27].

In the case of cessation of the injury, when the removal of cell debris and/or apoptotic bodies occurs, a switch from the Ly6C^high^ phenotype into Ly6C^low^ resolution macrophages can be observed. This means that Ly6C^low^ resolution macrophages release anti-inflammatory cytokines, mainly IL-10 and metalloproteases able to degrade, with time, the ECM in excess [13,16,17,25,26,27,61]. In addition, resolution macrophages can also induce apoptosis of HSC/MFs, facilitating fibrosis resolution [25,26,27,61].

Although the concept of fibrosis as a potentially reversible event has originated mostly from pre-clinical studies [61], it should be noted that reversion of fibrosis and regression of cirrhosis has also been documented in human patients, particularly in HBV and HCV chronic patients that displayed a sustained virological response (SVR) when treated with antiviral direct agents [62,63]. More recently, evidence of regression has been reported in NASH patients, achieving a significant reduction of body weight either as a consequence of lifestyle change or following bariatric surgery [64,65,66]. As a note of caution, it should be noted that in a limited number of NASH patients following bariatric surgery or HCV patients following therapy with direct antiviral agents, fibrosis has been reported to persist, and in few cases, to even progress [66,67].

## 4. ROS and Oxidative Stress in CLD Progression

### 4.1. The Impact of Oxidative Stress in CLDs: Introductory Remarks

Oxidative stress is a condition caused by an imbalance between an excess generation of ROS and the ability of antioxidant defenses, either enzymatic or non-enzymatic, to inactivate, eliminate, or scavenge these reactive products [68,69,70,71,72,73,74,75]. In a chronic injury environment, ROS can be generated, such as the superoxide anion (O_2_^•−^), hydrogen peroxide (H_2_O_2_), hydroxyl radical (^•^OH), as well as other redox-related reactive mediators of oxidative stress, such as the end-products of lipid peroxidation (LPO). LPO is a complex chain reaction initiated by ROS such as ^•^OH or other free radicals interacting with polyunsaturated fatty acids (PUFA) of membrane phospholipids that leads to the oxidative degradation of PUFA [68,69,70,71,72,73,74,75]. The end-products of LPO are mainly represented by either reactive aldehydes, such as malondialdehyde (MDA) and 4-hydroxy-2,3-alkenals (HAKs) of different chain lengths, or F_2_-isoprostanes. HAKs (mostly 4-hydroxy-2,3-nonenal or HNE) and F_2_-isoprostanes are relatively stable lipophilic compounds that can easily diffuse in the cellular environment and cross biological membranes, being able to exert both cytotoxic and signaling action [68,69,70,76]. The detection of these lipophilic compounds in a chronically injured liver or even biological fluids has been proposed as a reliable way to evaluate oxidative stress occurring in vivo.

Among ROS, particular mention is also due to nitric oxide or NO, a small hydrophobic molecule that, in addition to its general role in controlling the vascular tone, cellular adhesion, vascular permeability, and platelet adhesion, can also form the powerful pro-oxidant peroxynitrite (ONOO^−^) through the interaction with O_2_^•−^. Peroxynitrite, in turn, can potentially oxidize any cellular constituent, leading to the disruption of cell signaling pathways and the induction of hepatocyte injury and death [77].

In progressive CLDs, oxidative stress is believed to mainly represent the consequence of two events: (i) The direct impact of the etiological agent or condition on parenchymal cells, then resulting in increased intracellular generation (and release in the microenvironment following injury and death) of ROS and other redox-related reactive intermediates; (ii) the activation of innate immunity (i.e., inflammatory) cells following significant hepatocyte injury and death. The role of oxidative stress in contributing to fibrogenic progression may be influenced by the following aspects and issues [1,2,3,4,5,6,7,8,9,21,24,68,69,70,71,72,73,74,75,76]:

- Oxidative stress can per se contribute to hepatocyte injury and death, favoring the perpetuation of chronic liver injury and inflammatory response.
- ROS and some redox-related reactive mediators have been reported to be able to directly modulate the behavior of hepatic MFs, particularly HSC/MFs; this issue will be extensively described below (Section 4).
- An increased intracellular generation of ROS, directly related to fibrogenesis, has been described to specifically occur also in hepatic MFs as a consequence of the activation of NADPH oxidase isoforms in response to several peptide mediators as better described below.

### 4.2. A Synopsis of Critical Redox Events: From Cytotoxicity to Redox Signaling

Oxidative stress is considered a mechanism able to induce cell damage and death [68,69,70,71,72,73,74,75,76,77,78]. Under physiological conditions, antioxidant defenses, including enzymatic activities (i.e., superoxide-dismutase isoforms or SODs, catalase, glutathione peroxidase isoforms or GPXs, glutathione-disulfide reductase), specialized proteins such as thioredoxins, as well as naturally occurring molecules (i.e., α-tocopherol, reduced glutathione or GSH, β-carotene, ascorbate, urate) concur to maintain the redox homeostasis.

ROS can interact with any intracellular biological macromolecule leading to (i) the induction of lipid peroxidation of PUFA of phospholipids of biological membranes, PUFA degradation and fragmentation, and eventually, significant membrane injury; (ii) the inactivation of either structural or enzymatic proteins through the oxidation of –SH groups, as well as the formation of di-tyrosine, protein cross-linking, or intramolecular disulfide bonds; and (iii) oxidative damage to DNA, resulting in the formation of adducts or even strand breaks, events that can affect cell survival or even lead to mutation and, possibly, neoplastic transformation.

All the damaging reactions elicited by ROS, HAKs, and NO-derived intermediates concur to induce cell injury and the death of hepatocytes by significantly altering intracellular redox homeostasis, and can potentially elicit a condition of redox signaling [68,69,70,71,72,73,79].

**Figure 4:**
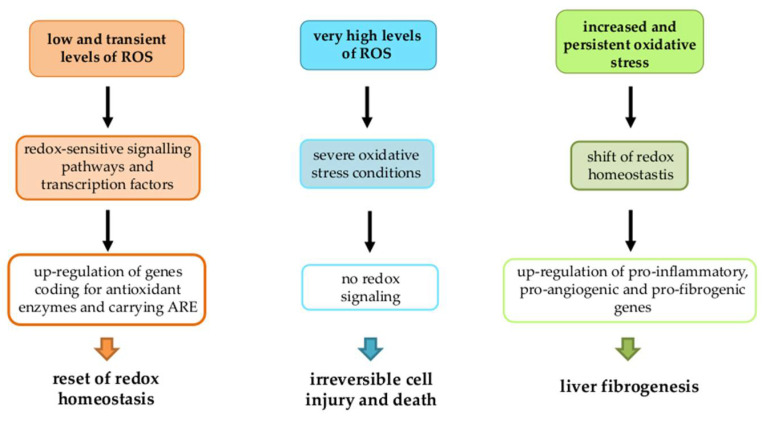
Three possible scenarios depending on intracellular ROS and other redox reactive intermediates levels.

Depending on intracellular ROS and other redox reactive intermediates levels, three main scenarios may occur (Figure 4):

1. Low and transient levels: Defined redox-sensitive signaling pathways and transcription factors lead to the up-regulation of genes coding for antioxidant enzymes and carrying ARE (antioxidant responsive element) sequences in order to reset redox homeostasis.
2. Very high levels: Typical of acute liver injury, these can lead to a condition of severe oxidative stress resulting in irreversible cell injury and death before any redox signaling may occur.
3. Increased and persistent oxidative stress: Typical of chronic liver injury and not able to induce cell death, this can lead to a shift of redox homeostasis to a chronically deregulated state. This, in turn, up-regulates different target genes (pro-inflammatory, pro-fibrogenic, pro-angiogenic, etc.) involved in CLD progression [61,62,80,81,82,83,84], making this latter scenario strongly related to liver fibrogenesis.

## 5. Hepatic MFs: When Redox Changes Modulate Phenotypic Responses

### 5.1. Oxidative Stress and HSC/MFs: From Induction of Cell Death to Survival

Concerning the ability of oxidative stress to model hepatic MFs responses, from only the early 2000s did researchers decide to analyze whether and how MFs, particularly HSC/MFs, may be affected by ROS in terms of cell injury and death as well as survival. HSC/MFs usually survive and operate their phenotypic responses during chronic liver injury and then oxidative stress but can rapidly undergo apoptosis during injury resolution. An initial study in 2004 reported that O_2_^•−^ could cause apoptosis of activated HSCs [85]. The authors suggested that O_2_^•−^ induced apoptosis by involving cytochrome c release, increased the expression of Bax, and, of course, the activation of executionary caspase 3, as well as the hydrolysis of polyADP-ribose polymerase; moreover, they demonstrated that the pro-apoptotic action of O_2_^•−^ was concentration-dependent in inhibiting DNA synthesis and reducing cell viability. However, in the same study, it was also proposed that O_2_^•−^ could up-regulate the expression of the antiapoptotic protein Bcl-xL and NF-kB transcriptional activity [85], two mechanisms potentially able to prevent or limit apoptosis. In a subsequent study, human-cultured HSC/MFs were exposed to controlled O_2_^•−^ generation rates in order to reproduce conditions detected in vivo, ranging from mild to moderate inflammation (0.8–1.2 nmol/min/mL). In these controlled conditions, human HSC/MFs were found to be extremely resistant to the induction of cell death, and only when very high levels of O_2_^•−^ were reached did HSC/MFs die either by apoptosis or necrosis/necroptosis, suggesting that HSC/MFs were able to easily survive even in quite severe conditions of oxidative stress [86]. By contrast, lower levels of ROS compatible with those detected in vivo in conditions of liver injury were able to elicit phenotypic responses in human HSC/MFs. These results confirmed previous data obtained in human HSC/MFs exposed to HNE. In this case, only very high and unrealistic HNE concentrations, unlikely to be reached in vivo, induced cell injury but not apoptotic cell death, whereas much lower levels of HNE induced selected phenotypic responses [87]. These in vitro studies suggested that human HSC/MFs easily survive ROS and HNE that are able, at lower and non-cytotoxic concentrations, to induce pro-fibrogenic phenotypic responses.

Why were human HSC/MFs so resistant to the induction of cell death by ROS and related reactive intermediates [43]? Chandrasekar Gandhi [85] was the first to show an up-regulation of Bcl-xL in HSC activated by O_2_^•^. This was an important step in understanding how HSCs survive ROS. Interestingly, another study confirmed that, in human HSC/MFs, Bcl-2 was also relevant in apoptosis control [43]. Morphological analyses showed that Bcl-2 was markedly over-expressed in HSC/MFs detected in liver specimens from patients with HCV advanced disease [43]. Data obtained in these different studies demonstrated that liver MFs, particularly human HSC/MFs, can survive ROS and other redox-related intermediates generated during the fibrogenic progression of CLD.

### 5.2. The Critical Pro-Fibrogenic Role of NADPH Oxidase of MFs

**Figure 5:**
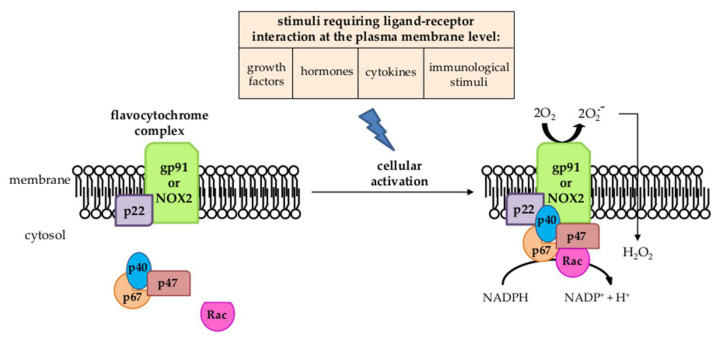
NADPH oxidase structure and activation.

In the scenario just described, the reader should keep in mind that the increased generation of intracellular ROS levels in hepatic MFs is due mainly to the activation of NADPH-oxidase (NOX) [2,3,4,6,7,8,70,88]. NOX (Figure 5) is a multi-subunit transmembrane complex that can generate either O_2_^•−^ or H_2_O_2_ in response to several stimuli requiring a ligand–receptor interaction at the plasma membrane level [89].

Following the ligand–receptor interaction by agonists, the cytosolic regulatory components of the NOX complex (p47^phox^, p40^phox^, p67^phox^, and Rac) translocate to the membrane-bound flavocytochrome complex (formed by the catalytic subunit gp91^phox^ or NOX2, the phagocytic form of NOX, and the regulatory subunit p22^phox^) to then operate the enzymatic activity [89]. The non-phagocytic NOXs in most cells replace NOX2 with other isoforms (NOX1, NOX3, NOX4, NOX5, DUOX1, and DUOX2). Concerning liver MFs, particularly HSC/MFs, they express different NOX isoforms, including the phagocytic NOX2 isoform, usually expressed by neutrophils, macrophages, and other innate immunity cells, as well as the NOX1 and NOX4 isoforms. The first study to describe the presence of NOX in HSC/MFs also suggested that the pro-fibrogenic action of Ang II was dependent on the concomitant activation of NOX and the related ROS-dependent activation of MAPKs, phosphorylation of c-Akt, and increased AP-1 DNA binding activity [90]. This was prevented by using either losartan, the inhibitor of Ang II type 1 receptor (AT1), or the NOX inhibitor diphenyl-phenyleneiodonium. In the following years, the role of NOX rapidly emerged in relation to the action of Ang II [90], but subsequent studies revealed that NOX activation was elicited by practically all the relevant pro-fibrogenic peptide ligands able to sustain MFs persistent activation, including PDGF, TGFβ1, VEGF-A, bFGF, ET-1, Ang II, and cytokines such as IL-1β, TNF, and IFN-γ, following the interaction with their cognate receptors [6,7,8,70,88]. Over the years, it became clear that the activation of NOX and the intracellular generation of ROS are common events in the modulation of the up-regulation of collagen type I expression in HSC/MFs [88], such as, for example, after engulfment by apoptotic bodies from dead hepatocytes [91,92]. Moreover, NOX and ROS are also involved in signaling pathways (MAPK cascades, NF-kB system, PI3K/Akt signaling, etc.) and in other MF-dependent pro-fibrogenic responses such as oriented migration, ECM synthesis and remodeling, proliferation, and contractility [88].

Concerning this point, in the next section of the review, the role of ROS as pro-fibrogenic mediators will be described.

### 5.3. ROS and Oxidative Stress-Related Intermediates as Pro-Fibrogenic Mediators

Many pre-clinical studies showed that antioxidant supplementation can significantly prevent CLD progression in animal models by preventing or reducing in vivo oxidative stress and/or lipid peroxidation [7,8,69,70,93], suggesting a pro-fibrogenic role of oxidative stress, ROS, and other redox-related reactive intermediates. Although few of these pre-clinical approaches were found to be effective when translated into clinical trials in humans, they indicated that ROS and other redox-related reactive intermediates can significantly modulate MFs phenotypic responses.

The exposure of rodent and human HSC/MFs to ROS or other redox-dependent reactive intermediates results in increased expression of ECM components. An initial study, performed on human HSC/MFs exposed to very low levels of HNE or to conditions leading to lipid peroxidation, showed a strong up-regulation of pro-collagen type I [94], a finding confirmed by subsequent studies employing other 4-hydroxy-2,3-alkenals of different chain lengths [95], as well as the other end-products of lipid peroxidation such as MDA [96] or F_2_-isoprostanes [97]. Along these lines, the same results were obtained when rat or human HSC/MFs were exposed to extracellularly available ROS such as H_2_O_2_ or O_2_^•−^ released by activated neutrophils, generated by the xanthine/xanthine-oxidase system, or by exposing HSC/MFs to the conditioned medium of normal hepatocytes undergoing oxidative stress [98,99,100].

Another experimental procedure, resulting in the redox-dependent up-regulation of pro-collagen type I in HSC/MFs, was to co-culture these cells with hepatocytes transfected to over-express the ethanol metabolizing enzyme cytochrome P450 2E1 (CYP 2E1) and then to expose cells to ethanol resulting in concomitant CYP 2E1-dependent ROS generation [8]. Other studies adopted the strategy of transfecting rat HSC/MFs to express human CYP 2E1, showing the up-regulation of pro-collagen type I transcription and synthesis. This was proportional to CYP 2E1 levels in HSC/MFs and exacerbated by CYP 2E1 increased generation of ROS following the exposure of cells to ethanol or arachidonic acid [101,102,103]. Different approaches were then adopted to understand how ROS and end-products of lipid peroxidation, released in the extracellular environment by injured hepatocytes and able to cross the membrane of cells, may affect the release of pro-collagen I from HSC/MFs. Concerning, for example, HNE, this molecule is able to elicit in HSC/MFs a transient activation of JNK isoforms with consequent nuclear translocation, up-regulation of c-Jun, and increased AP-1 binding to DNA [104], a mechanism very close to that identified in rat HSC/MFs exposed to UV irradiation [105]. Another study showed that TGFβ1, the most potent pro-fibrogenic cytokine, up-regulated collagen type I in HSC/MFs by stimulating H_2_O_2_-dependent signaling that involved the binding of the p35 C/EBPβ protein to the promoter of the collagen α1(I) gene [100], a mechanism related to the modulation of intracellular levels of GSH [106] or the involvement of p38MAPK [107]. Similarly, the H_2_O_2_-dependent involvement of the C/EBPβ protein was also found to mediate the acetaldehyde-dependent up-regulation of collagen type I [108] as well as, in part, TGFβ1 [109]. Moreover, intracellular generation of H_2_O_2_ was also reported to mediate leptin-induced α1(I) collagen gene expression in LX2 immortalized human HSCs through signaling involving Janus kinases 1 and 2 (JAK1 and JAK2) as well as Erk1/2 and p38MAPK [110]. From these studies and many others not mentioned here, the reader can take home this message: Independently of the origin, extra- or intra-cellular, ROS and other intermediates can efficiently mediate the signaling of HSC/MFs and ECM deposition, exerting pro-fibrogenic effects.

In some of the pioneer studies on the role of ROS in modulating the behavior of HSC/MFs, it was proposed that ROS may also mediate or contribute to the process of activation/transdifferentiation and/or proliferation. An initial indication was provided by an elegant study performed on HSC/MFs co-cultured with HepG2 cells manipulated to overexpress CYP 2E1 and then raise the generation of ROS. In these settings, HSC/MFs started to significantly increase the expression of α-SMA, a marker of MF differentiation, and to actively proliferate [111]. The involvement of ROS was also deduced from the fact that the treatment of HSC/MFs with antioxidants resulted in prevention of these responses, particularly of proliferation, in response to PDGF [112,113], likely through NOX activation [114], as also reported for Ang II-induced proliferation [90]. The mitogenic effect of ROS was referred to as the ability to interfere with a critical cysteine residue in Raf-1, MEK, and Erk signaling elements, as also suggested by the fact that N-acetyl-cysteine resulted in HSC/MFs cell cycle arrest in the G1 phase [113].

The proliferation of MFs was not observed in the presence of other reactive intermediates such as HNE and HAKs of different chain lengths, which result as ineffective in stimulating the proliferation of human HSC/MFs at concentrations compatible with those detected in vivo [115,116]. Moreover, these aldehydic mediators inhibited DNA synthesis elicited by PDGF-BB by selectively inhibiting PDGF-β receptor intrinsic tyrosine kinase activity and downstream signaling pathways [115,116]. This effect was transient, and the sensitivity of HSC/MFs to PDGF-BB, through subsequent up-regulation of the expression of PDGF-Rβ, was recovered within 48 h, similarly to what was shown for cells exposed to very high levels of O_2_^•−^ or H_2_O_2_ [86,115,116]. The different effect of HNE and HAKs is likely to depend on the peculiar mechanism of action of these aldehydes that operates by forming adducts to proteins by means of nucleophilic Michael-type reactions [117,118], as shown in the case of JNK activation [104]. In addition, HNE, differently from ROS, cannot activate NF-kB in HSC/MFs [116,117,118], but rather can even inhibit c-Myb, which has been proposed to play a role in ROS-mediated proliferation [119].

As an additional difference, HNE has been described to act as a pro-fibrogenic stimulus only on fully activated HSC/MFs [87,118] but not on quiescent HSC or primary HSC at a very early stage of culture, which is different from that reported for ROS [2,3,4,6,7,8,70]. This is likely the consequence of the fact that quiescent HSC can eliminate H_2_O_2_ less efficiently than fully activated HSC/MFs [102,106]. By contrast, HSC/MFs are more sensitive to HNE since they do not express significant levels of aldehyde dehydrogenase and glutathione-S-transferase isoforms necessary to efficiently remove HNE [104,120]. Overall, HNE has been reported, differently from ROS, to up-regulate a limited list of pro-fibrogenic genes such as those encoding for collagen type I, TGFβ1, and TIMP-1 [8,70,87,118].

Another peculiar difference between ROS and HNE relies on the fact that HNE does not affect chemotaxis [86,87], whereas two different laboratories have shown the ability of O_2_^•−^ generated in the extracellular environment to induce oriented migration of HSC/MFs by stimulating the Ras/Erk pathway [86,121], while chemotaxis was not stimulated by adding H_2_O_2_ extracellularly [86]. In one of these studies, O_2_^•−^ was also found to promote the invasiveness of HSC/MFs, that is, oriented migration in matrigel, and this event was related to the ability of O_2_^•−^ to up-regulate the expression of MMP-2 [121].

These studies on the oriented migration of HSC/MFs by ROS were rapidly implemented by several others that showed unequivocally that intracellular generation of ROS significantly contributed to chemotaxis induction by peptide chemoattractants. The first study to be mentioned reported that Ang II [90] stimulated chemotaxis through the involvement of NOX isoforms, as shown by using modified Boyden chambers and in vitro wound-healing assays, once again confirming previous data indicating the critical role of NOX in mediating HSC/MFs behavior [114]. A subsequent study confirmed and extended this issue by showing that HSC/MFs were induced to migrate by a panel of polypeptide chemoattractants including PDGF, VEGF, CCL2, and Ang II that were all able to induce an NADPH-oxidase-dependent intracellular rise in ROS, resulting in the activation of ERK1/2 and JNK1/2 and then of HSC/MFs migration [44]. In addition, in the study, two pro-oxidant molecules such as menadione or 2,3-dimethoxy-1,4-naphthoquinone were also used, which can generate the intracellular superoxide anion or hydrogen peroxide, respectively, in the absence of NOX involvement. The treatment of HSC/MFs with these molecules once again resulted in the activation of ERK1/2 and JNK1/2 and in the induction of migration. Specific silencing of the two isoforms indicated that the JNK1 isoform was predominant in sustaining migration. The overall message from this study was quite clear: Intracellular generation of ROS by itself, whether dependent on NOX involvement or not, is sufficient to induce HSC/MFs migration.

The involvement of the intracellular generation of ROS was further confirmed in more recent years as a critical issue in mediating the response of HSC/MFs to a number of additional polypeptide mediators [7,8,9]. Since, in CLD progression, hypoxia is an important condition favoring liver fibrogenesis, here we mention some studies with a specific focus on mediators regulated by hypoxia-inducible factors (HIFs) [8,9,122]. Among these mediators, SerpinB3 is a serine protease inhibitor whose expression was shown to be regulated by hypoxia- and, specifically, HIF-2α [123], and was overexpressed in both fibrotic/cirrhotic human liver specimens and animal models of CLD [80]. SerpinB3 was reported to strongly up-regulate the expression of several genes involved in fibrogenesis and promote oriented migration, but not cell proliferation, in both human HSC/MFs or LX2 cells [80]. In these MF-like cells, human recombinant SerpinB3 both increased migration and intracellular ROS levels; once again, migration was critically dependent on intracellular ROS and activation of JNK1/2, being almost abolished by pre-treatment with pharmacological inhibitors of either NADPH-oxidase or JNK1/2 [80]. Another example of an HIF-related pro-fibrogenic mediator acting through intracellular ROS generation is represented by oncostatin M (OSM), a cytokine belonging to the IL-6 family, which is known to orchestrate hypoxia-modulated hepatic processes involving HIF-1. Recently, OSM has been reported to be up-regulated in the liver of either NASH patients or of mice fed a NASH-inducing diet [81], and human recombinant OSM was found to stimulate the migration of human LX2 cells in both Boyden’s chambers and wound healing assays. OSM-induced migration again involved intracellular ROS generation and the activation of Ras/Erk, JNK1/2, and PI3K/Akt, as shown for other chemoattractants, but also STAT1/STAT3 pathways and HIF-1α. In particular, OSM-induced migration was suggested to depend on a biphasic mechanism requiring the early intracellular generation of ROS and late HIF1-dependent expression and release of VEGF, indirectly suggesting that OSM may play a role also in sustaining angiogenesis in a redox-dependent way [81]. This was not surprising since another polypeptide mediator, such as leptin, was found to modulate the angiogenic properties of HSC [82,83]. In an initial study, it was shown that leptin was able to increase gene expression of the pro-angiogenic cytokines VEGF and Ang- 1 in human HSC/MFs. Leptin was also found to increase the abundance of HIF-1, which regulates angiogenic gene expression, in an ERK- and PI3K-dependent way [82]. In a subsequent study, designed to analyze these pathways in detail, Leptin was found to activate the mammalian target of the rapamycin (mTOR) pathway, and homologous results were observed when HSC/MFs were exposed to PDGF-BB [83]. Of interest, both leptin and PDGF-BB increased the expression of HIF-1α and VEGF in HSC/MFs. Dedicated experiments indicated that the up-regulation of VEGF by both leptin and PDGF-BB involved mTOR activation and increased intracellular generation of ROS in a NOX-dependent manner. However, the induction of HIF-1α required NOX but not mTOR activation [84].

## 6. Antioxidant as a Therapy in Liver Fibrosis

From the data reported here, a scenario emerged in which ROS could represent one of the targets for the treatment of liver fibrosis. Antioxidant therapy, by direct free radical scavenging or enhancing the endogenous antioxidant machinery, has been shown to be effective in most cases in preventing/attenuating experimental fibrosis [124]. Indeed, decreasing free radicals within the hepatic parenchyma has been proposed as a potential suitable, safe, and inexpensive therapeutic strategy against fibrosis.

As previously mentioned, one of the major sources of increased intracellular ROS concentrations is represented by different NOX subtypes. This background of findings provided the rationale to design and test putative antifibrotic therapeutic strategies selectively targeting NOX as a possible and more specific alternative to the use of antioxidants, with the latter being effective in preclinical models but mostly ineffective in clinical trials [125,126].

Along these lines, pre-clinical studies reported that the dual NOX1/4 inhibitor GKT137831 was able to inhibit specific pathways and responses in cultured HSC/MFs. GKT137831 not only suppressed ROS production but also prevented HSC activation via the inhibition of inflammation- and proliferation-associated signaling in two different murine models of CLD [84].

Concerning compounds with antioxidant properties that seem to be useful to improve liver function and reverse fibrosis, Table 2 offers an overview of potential therapeutic drugs/molecules that directly target the molecular pathways responsible for ROS generation.

However, the efficacy of these compounds is still controversial. Although in pre-clinical studies antioxidant supplementation (for example vitamin E) has been reported to be effective at preventing cell death, inflammatory response, and liver fibrosis, these strategies were found to be less effective in clinical trials on human patients (particularly in the prevention of fibrogenic progression) [7,69,70]. Literature data obtained from patients reported that specific genotypes of antioxidant and pro-oxidant genes are associated with higher susceptibility to developing liver cirrhosis and hepatocellular carcinoma while other individual characteristics (age, metabolomic profiling) can influence the efficacy of antioxidants on CLD [135]. These considerations suggest the need for additional research to establish the safety, efficacy, and dosage of antioxidants as well as the eligible patient profile for antioxidant treatment.

The data here reported suggest that novel promising strategies for the treatment of liver fibrosis may be represented by the combined application of compounds able to promote antioxidant responses as well as to modulate targets involved in hepatocyte protection, HSC activation and immune modulation [136].

## 7. Concluding Remarks

An impressive amount of literature data indicates that hepatic MFs represent a unique cellular phenotype that plays a critical role in driving liver fibrogenesis during CLD progression. In a fashion similar to that reported for MFs involved in other types of chronic injury and organ fibrosis [7,8], hepatic MFs, regardless of the cellular origin, exhibit a rather common pattern of pro-fibrogenic phenotypic responses, which are mostly elicited or sustained by either ROS and other oxidative stress-related reactive intermediates or a plethora of mediators (including growth factors, cytokines, chemokines, adipokines, and others) that often operate through the up-regulation of intracellular generation of ROS.

In the present review, we have tried to offer an overview of the role of MFs in the fibrogenic progression of CLD with a focus on the direct or indirect role of ROS and other redox-related mediators in modulating pro-fibrogenic phenotypic responses operated by these peculiar cells. Although more research is needed to elucidate several still incompletely investigated mechanistic aspects, we would like to conclude this review by suggesting that the final pro-fibrogenic response of hepatic MFs to ROS and oxidative stress mediators should be envisaged as a relatively unpredictable one, being significantly affected by the integration of several issues, including at least the following: (i) The steady-state concentration of reactive species under analysis; (ii) the intrinsic state of the target cells (that is, activated versus quiescent); and (iii) the presence of growth factors, cytokines, and other mediators in the extracellular microenvironment or of other cellular sources of ROS or HNE.

## Figures and Tables

**Table 1:** Potential cellular origin of hepatic myofibroblasts and related biomarkers.

Potential Cellular Origin of Hepatic Myofibroblasts	Biomarkers
**Hepatic stellate cells (HSCs)** 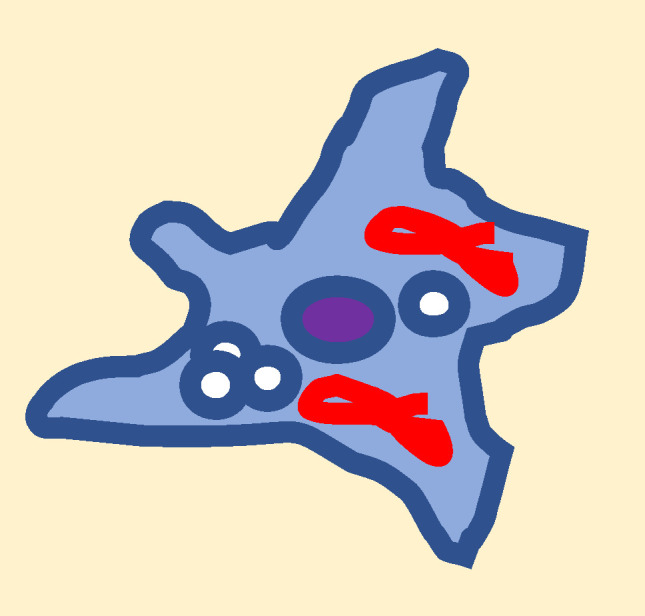	- glial fibrillary acidic protein (GFAP) - plateled-derived growth factor receptor β (PDGF β) - nerve growth factor receptor p75 subunit - lecithin-retinol acyltransferase (LRAT) - integrin ανβ3 - desmin - vimentin - mannose 6-phosphate/insulin-like growth factor II receptor - cytoglobin
**Portal fibroblasts (PFs)** 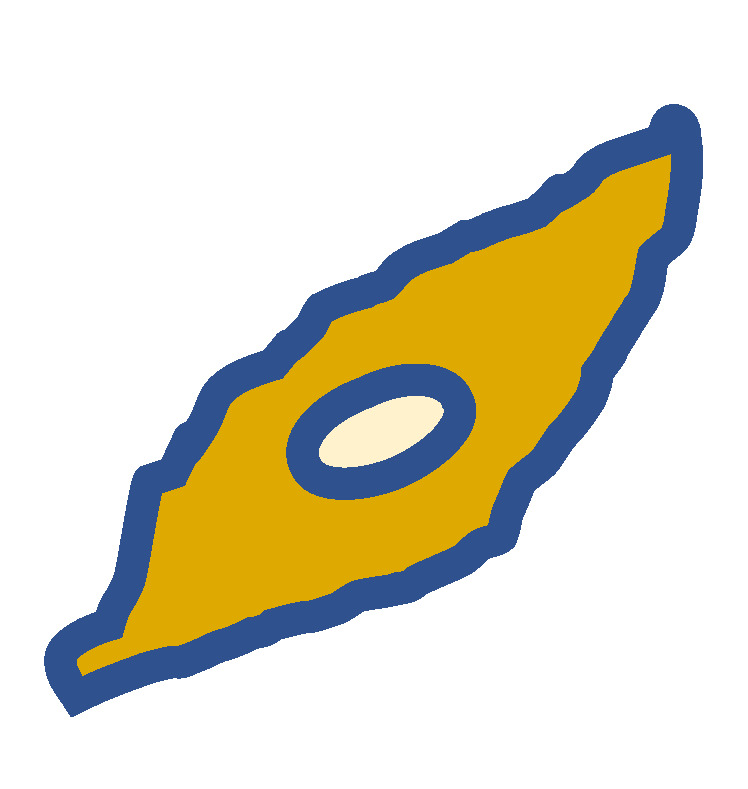	- α-SMA - ecto-ATPase nucleoside triphosphate - diphosphohydrolase-2 (NTPD2) - fibulin 2 - elastin - IL-6 - cofilin
**Mesenchymal stem cells** 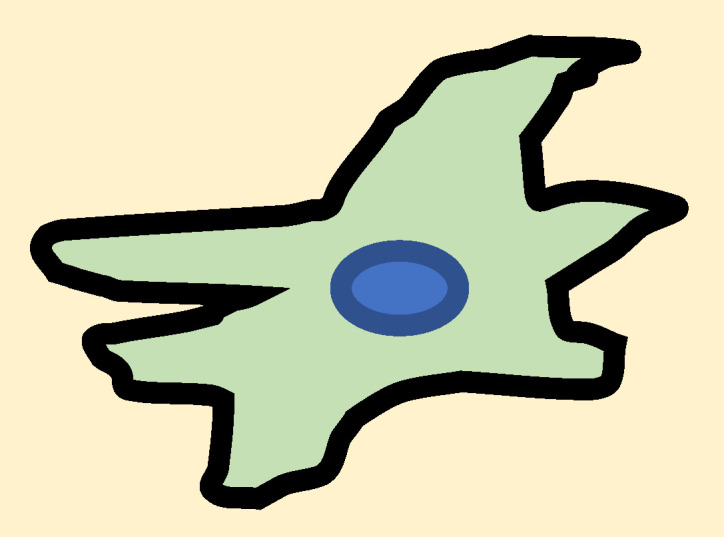	- CD90 - CD105 - CD29 - CD73

**Table 2:** Overview of novel potential therapeutic drugs that directly target the molecular pathways responsible for ROS generation.

Molecule	Target	Model	Effect	References
**Vitamin E**	ROS	NASH	protection of structural components of cell membrane from peroxidation	[127]
**Silibin**	ROS	ALD	increased of GSH concentration	[128]
**Chlormethiazole**	CYP2E1	ALD	reduction of proteasome proteolytic enzyme activity induced by ethanol	[129]
**Nrf2 activators**	Nrf2	NAFLD/NASH	prevention of inflammation, trygliceride accumulation	[130,131]
**Ethyl pyruvate**	Nrf2	ALD	increase of anti-inflammatory factors	[132]
**MCC950**	NLPR3	NASH	decrease of AST and ALT and liver inflammation	[133,134]

## Abbreviations

α-SMA: α-smooth muscle actin; ALD: alcoholic liver disease; AT1: Ang II type 1 receptor; bFGF: basic fibroblast growth factor; CLD: chronic liver disease; CTGF: connective tissue growth factor; CYP 2E1: cytochrome P450 2E1; DAMPs: damage-associates molecular patterns; ECM: extracellular matrix; EGF: epidermal growth factor; EMT: epithelial-to-mesenchymal transition; ET-1: endothelin-1; EVs: extracellular vesicles; GFAP: glial fibrillary acidic protein; GPXs: glutathione peroxidase isoforms; GSH: reduced glutathione; H2O2: hydrogen peroxide; HAKs: 4-hydroxy-2,3-alkenals; HCC: hepatocellular carcinoma; HGF: hepatocyte growth factor; HIFs: hypoxia-inducible factors; IGF: insulin-like growth factor; IL-1ra: IL-1 receptor antagonist; IRE1α: inositol-requiring enzyme 1α; JAK1: Janus kinases 1; JAK2: Janus kinase 2; KCs: Kupffer cells; LPO: lipid peroxidation; LRAT: lecithin-retinol acyltransferase; MARCO: alveolar macrophage marker gene; MBOAT7: membrane bound O-acyltransferase domain-containing 7 gene; MDA: malondialdehyde; MFs: myofibroblasts; MoMΦs: monocyte-derived macrophages; mTOR: mammalian target of rapamycin; NAFLD: non-alcoholic fatty liver disease; NK: natural killer cells; NKT: natural killer T cells; NOX: NADPH-oxidase; NTPD2: nucleoside triphosphate diphosphohydrolase-2; O_2_^•−^: superoxide anion; ONOO-: peroxynitrite; ^•^OH: hydroxyl radical; OSM: oncostatin M; PAMPs: pathogen-associated molecular patterns; PDGFRβ: platelet-derived growth factor receptor β; PERK: PKR-like endoplasmic reticulum kinase; PNPLA3: patatin-like phospholipase domain containing-3; PRR: pattern-recognition receptor; PUFA: polyunsaturated fatty acids; ROS: reactive oxygen species; SECs: sinusoidal endothelial cells; SODs: superoxide-dismutase isoforms; TIMPs: tissue inhibitors of metalloproteases; TM6SF2: transmembrane 6 superfamily member 2 gene; TMC4: transmembrane channel-like 4 gene.
